# Posterior hemivertebra resection and monosegmental fusion in the treatment of congenital scoliosis

**DOI:** 10.1308/003588414X13824511650173

**Published:** 2014-01

**Authors:** X Zhu, X Wei, J Chen, C Li, M Li, Y Qiao, B Ran

**Affiliations:** ^1^Department of Orthopedic, Changhai Hospital, The Second Military Medical University, Shanghai,China; ^2^Department of Orthopedics, The Affiliated Hospital of Xuzhou Medical College, Xuzhou, Jiangsu Province,China; ^3^Department of Orthopedics, Kunming General Hospital of Chengdu Military Command, Kunming,China; ^4^Xinhua Hospital affiliated to Shanghai Jiaotong University School of Medicine Chongming Branch, Shanghai,China; ^5^Department of Otorhinolaryngology, The Affiliated Hospital of Xuzhou Medical College, Xuzhou, Jiangsu Province,China; ^6^The Second People’s Hospital of Lian-yun-gang, Lian-yun-gang, Jiangsu Province,China

**Keywords:** Hemivertebra resection, Monosegmental fusion, Congenital scoliosis

## Abstract

**INTRODUCTION:**

Posterior hemivertebra resection combined with multisegmental or bisegmental fusion has been applied successfully for congenital scoliosis. However, there are several immature bones and their growth can be influenced by long segmental fusion in congenital patients. Posterior hemivertebra resection and monosegmental fusion was therefore suggested for treatment of congenital scoliosis caused by hemivertebra.

**METHODS:**

Between June 2001 and June 2010, 60 congenital scoliosis patients (aged 2–18 years) who underwent posterior hemivertebra resection and monosegmental fusion were enrolled in our study. A standing anteroposterior x-ray of the whole spine was obtained preoperatively, postoperatively and at the last follow-up appointment to analyse the Cobb angle in the coronal and sagittal planes as well as the trunk shift.

**RESULTS:**

The mean preoperative coronal plane Cobb angle was 41.6º. This was corrected to 5.1º postoperatively and 5.3º at the last follow-up visit (correction 87.3%). The compensatory cranial curve was improved from 18.1º preoperatively to 7.1º postoperatively and 6.5º at the last follow-up visit while the compensatory caudal curve was improved from 21.5º to 6.1º after surgery and 5.6º at the last follow-up visit. The mean sagittal plane Cobb angle was 23.3º before surgery, 7.3º after surgery and 6.8º at the last follow-up visit (correction 70.1%). The trunk shift of 18.5mm was improved to 15.2mm.

**CONCLUSIONS:**

Posterior hemivertebra resection and monosegmental fusion seems to be an effective approach for treatment of congenital scoliosis caused by hemivertebra, allowing for excellent correction in both the frontal and sagittal planes.

A hemivertebra is the most common anomaly causing congenital scoliosis.^1,2^ Hemivertebra may occur at ipsilateral adjacent levels of the spine, which produces significantly asymmetrical spine growth, or one hemivertebra may be counterbalanced by another hemivertebra on the contralateral side of the spine in the same region, separated by one or several healthy vertebrae (termed as hemimetameric shift).^3^ The hemivertebra resection is performed initially using a simultaneous anterior and posterior approach, either in a one-stage or two-stage operation.^4,5^

With the development of the pedicle screws technique, hemivertebra resection can be performed successfully with only a posterior approach using a one-stage procedure.^6,7^ This can be performed with an excellent outcome, little injury and fewer complications.^8^ Recently, there have been reports on hemivertebra resection with multisegmental^9^ or bisegmental posterior instrumentation fusion^10^ for treatment of congenital scoliosis. However, in congenital patients, there are several immature bones and their growth can be influenced by long segmental fusion.^11^ The fusion scope should therefore be deceased and monosegmental fusion is advocated. To date, there have been no large series on posterior hemivertebra resection with monosegmental fusion. A retrospective study was performed to evaluate the surgical outcomes of posterior hemivertebra resection with only monosegmental fusion for congenital scoliosis caused by hemivertebra.

## Methods

All applicable institutional and governmental regulations concerning the ethical use of human volunteers were followed during the course of this research. From June 2001 to June 2010, 60 patients (30 male and 30 female) with congenital scoliosis caused by hemivertebrae underwent posterior hemivertebra resection and monosegmental fusion. Their mean age was 7 years (range: 2–18 years). Hemivertebrae located at the thoracic level were observed in 16 patients, at the thoracolumbar level in 37 patients and at the lumbar level in 7 patients. Of these patients, 46 were included in the study with fully segmented, 10 with partially segmented and 4 with unsegmented hemivertebrae. All the operations were performed by the same doctor.

### Preoperative examination

All patients received a standing anteroposterior x-ray examination of the whole spine, computed tomography (CT) of the whole spine and three-dimensional reconstruction to confirm the spinal deformity. Nervous system magnetic resonance imaging (MRI) was performed to test whether spinal intramedullary disease existed. Pulmonary function and ultrasonic cardiography were examined to evaluate whether the patients had lung or heart diseases.

### Operation technique

All patients underwent general anaesthesia and were placed in a prone position on a surgical table. Using a standard posterior approach, subperiosteal dissection was performed to expose the hemivertebra as well as the vertebra above and the vertebra below. Gauge 20 needles were inserted into the pedicles of the hemivertebra and the adjacent vertebrae. Fluoroscopy was used to confirm the hemivertebra and the pedicle screws were inserted after tapping.

The posterior elements of the hemivertebra including the lamina, spinous process, articular process and transverse process were removed to expose the pedicle, and the nerve roots above and below. In the thoracic spine, the rib head and the proximal part of the surplus rib on the convex side were exposed and resected. The anterior part of the hemivertebra was exposed by blunt dissection. Following this, a wedge osteotomy was performed from the convex to the concave side. Using a rongeur, an osteotome, a curette and abrasive drilling, the anterior vertebral body was hollowed. A thin layer of bone was residual in the anterior spinal cord; this was subsequently compressed gently.

After the anterior bone was removed using a rongeur, the hemivertebra wedge-shaped bone surface was achieved and gradually closed. Autogenous bone from the hemivertebra was used for posterolateral fusion and negative pressure drainage was retained. Intraoperative neurophysiological monitoring including somatosensory and motor evoked potentials was performed to identify and prevent injury to neurovascular structures during surgery. There was no difference in the surgical method for various types of hemivertebra.

### Postoperative management

Antibiotics were administered for three to five days following surgery. The wound drainage tube was removed at 48–72 hours after the operation. The patients were advised to walk and wear the brace for at least 6 months after the drainage tube was removed. All patients were followed up at 3 months, 6 months, 12 months and then every 6 months after surgery.

### Evaluation

All patients underwent standing anteroposterior x-ray examination of the whole spine preoperatively, postoperatively and during the follow-up period. The Cobb angles in the coronal and sagittal planes were determined. The compensatory cranial and caudal curve, and the trunk shift (defined as the perpendicular distance from the sacrum centre to the plumb line drawn from the midpoint of the C7 vertebra body) were also measured. In addition, the operative time, bleeding volume and complications were recorded.

## Results

The mean operation time was 172.7 minutes (range: 130–370 minutes), the mean bleeding volume was 350.5ml (range: 100–800ml) and the mean follow-up duration was 50.1 months (range: 24–110 months). The mean preoperative coronal plane Cobb angle of 41.6º was corrected to 5.1º postoperatively and 5.3º at the last follow-up visit (correction 87.3%). The mean compensatory cranial curve was improved from 18.1º preoperatively to 7.1º postoperatively and 6.5º at the last follow-up visit (correction 68.3%). The mean compensatory caudal curve was improved from 21.5º preoperatively to 6.1º postoperatively and 5.6º at the last followup visit (correction 73.3%). The mean sagittal plane Cobb angle was 23.3º before surgery, 7.3º after surgery and 6.8º at the last follow-up visit (correction 70.1%). The preoperative average trunk shift was 18.5mm. This was corrected to 15.2mm postoperatively and 10.1mm at the last follow-up visit (Table 1).

**Table 1:** Correction outcome in the coronal and sagittal planes

	Preoperative	Postoperative	Final follow-up	Correction ratio
Segmental scoliosis	41.6º	5.1º	5.3º	87.3%
Trunk shift	18.5mm	15.2mm	10.1mm	49.1%
Cranial compensatory bending	18.1º	7.1º	6.5º	68.3%
Caudal compensatory bending	21.5º	6.1º	5.6º	73.3%
Segmental kyphosis	23.3º	7.3º	6.8º	70.1%

Pedicle screw cut-out and deformity recurrence occurred in three patients, who needed further revision procedures. Rod breakage was noted in two patients. This was solved by implanting a new rod after two years. Two cases had delayed wound healing but the wounds did heal after regular dressing changes.

**Figure 1:**
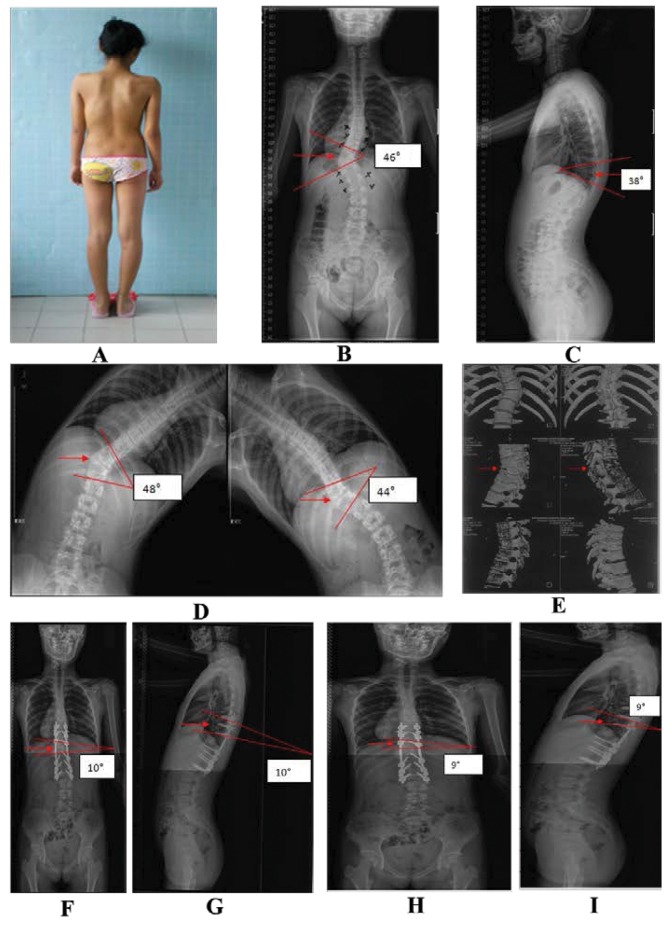
Images of a 12-year-old girl who underwent hemivertebra resection and monosegmental fusion for T11 hemivertebra deformity: Preoperative gross inspection (A); Preoperative anteroposterior x-ray indicating T11 hemivertebra deformity (arrow) with the scoliosis convex to the left and concave to the right, and a scoliosis Cobb angle of 46º (B); Preoperative lateral x-ray indicating T11 hemivertebra deformity (arrow) with a kyphosis Cobb angle of 38º (C); Preoperative left and right bending x-rays indicating T11 hemivertebra deformity (arrow) with a kyphosis Cobb angle of 48º and 44º (D); Preoperative computed tomography indicating T11 hemivertebra deformity (arrow) (E); Postoperative anteroposterior x-ray indicating the T11 hemivertebra had been resected (arrow) and good correction of the spine deformity had been achieved with a scoliosis Cobb angle of 10º (F); Postoperative lateral x-ray indicating the T11 hemivertebra had been resected (arrow) and good correction of the spine deformity had been achieved with a kyphosis Cobb angle of 10º (G); Anteroposterior x-ray 3 years following surgery indicating the T11 hemivertebra had been resected (arrow) with a scoliosis Cobb angle of 9º (H); Lateral x-ray 3 years following surgery indicating the T11 hemivertebra had been resected (arrow) with a kyphosis Cobb angle of 9º (I).

A typical hemivertebra resection case is shown in Figure 1.

## Discussion

Whether congenital scoliosis should be treated depends on the degree of deformity and the development tendency.^12^ Owing to the vigorous growing potential in hemivertebrae in children (especially fully segmented non-incarcerated hemivertebrae), the scoliosis deformity would be aggravated increasingly with the growth and development of the patient.^2^ This would increase the number of fusion segments and the difficulty in performing surgery. Consequently, the earlier the intervention begins, the better the effect on the patient. For example, Ruf and Harms reported that 15 of 28 congenital scoliosis patients with a mean age of 3 years and 4 months (range: 1–6 years) were treated by hemivertebra resection and monosegmental fusion of the adjacent vertebra.^13^ The compensatory cranial curve correction ratio was 78%, the compensatory caudal curve correction ratio was 65% and the segmental kyphosis Cobb angle correction ratio was 63%.

In our study, the mean age was 7 years (range: 2–18 years). All of these patients underwent posterior hemivertebra resection and monosegmental fusion. After the operation, the main curve Cobb angle correction ratio was 87.3%, the compensatory cranial curve correction ratio was 68.3% and the compensatory caudal curve correction ratio was 73.3%. The trunk shift was improved from 18.5mm preoperatively to 10.1mm postoperatively. The kyphosis Cobb angle correction ratio was 70.1% in the sagittal plane. These findings therefore demonstrate that posterior hemivertebra resection and monosegmental fusion in the treatment of congenital scoliosis in the early stages can result in a good outcome and can decrease development of the deformity.

Owing to osteoporosis and the immature vertebral endplates in children, the fixation pullout strength is low and the fixation would become flexible, leading to more complications related to internal fixation.^14^ Ruf *et al* reported that 8 of 41 patients (19.5%) showed postoperative complications including loosening of internal fixation in 3 patients, pedicle screw breakage in 3 patients and new deformity in 2 patients.^15^ All of the above patients had revision operations. In the study by Zhang *et al*, 56 patients (aged 1.5–17 years) underwent one-stage posterior hemivertebra resection with transpedicular instrumentation.^16^ There was one case of delayed wound healing, two fractures of the pedicle at the instrumented level, two cases of rod breakage and one proximal junction kyphosis that required revision. In our study, fixation breakage was observed in five patients and delayed healing was noted in two.

According to our experience, one should pay more attention to the following aspects during the process of posterior hemivertebra resection and monosegmental fusion:

- Pedicle anatomy should be analysed accurately using x-ray, CT, MRI or neural electrophysiology to minimise pedicle injury. Pedicle screws should be implanted only once if possible as repeated implantation could damage the pedicle and the pullout strength will be decreased.
- Hemivertebrae should be resected thoroughly to avoid the residual tissue enlarging the pedicle screw strength.
- If there is a larger space after hemivertebra resection or obvious kyphosis, a titanium cage should be implanted for the anterior reconstruction to increase the anterior stability.
- If a vertebra block or rib deformity exists, a concave release operation should be carried out to decrease the pedicle screw strength.
- Vertebra fusion should be promoted to avoid non-union by implanting autogenous or allogeneic bone. As expected, no non-union was experienced in our study as autogenous or allogeneic bone grafting were adopted.

## Conclusions

Posterior hemivertebra resection and monosegmental fusion is effective in treating congenital scoliosis. It can correct the deformity, and retains more active segments and growth potential in children, even in infants. Nevertheless, fixation complications are still one of the biggest challenges with this technique and this needs more attention in future studies.
